# ADARp150 counteracts whole genome duplication

**DOI:** 10.1093/nar/gkae700

**Published:** 2024-08-27

**Authors:** Frank van Gemert, Alexandra Drakaki, Isabel Morales Lozano, Daniël de Groot, Maud Schoot Uiterkamp, Natalie Proost, Cor Lieftink, Marieke van de Ven, Roderick L Beijersbergen, Heinz Jacobs, Hein te Riele

**Affiliations:** Division of Tumor Biology and Immunology, The Netherlands Cancer Institute, Amsterdam, The Netherlands; Division of Tumor Biology and Immunology, The Netherlands Cancer Institute, Amsterdam, The Netherlands; Division of Tumor Biology and Immunology, The Netherlands Cancer Institute, Amsterdam, The Netherlands; Division of Tumor Biology and Immunology, The Netherlands Cancer Institute, Amsterdam, The Netherlands; Division of Tumor Biology and Immunology, The Netherlands Cancer Institute, Amsterdam, The Netherlands; Mouse Clinic for Cancer and Aging Research, Preclinical Intervention Unit, The Netherlands Cancer Institute, Amsterdam, The Netherlands; Division of Molecular Carcinogenesis, NKI Robotics and Screening Center, The Netherlands Cancer Institute, Amsterdam, The Netherlands; Mouse Clinic for Cancer and Aging Research, Preclinical Intervention Unit, The Netherlands Cancer Institute, Amsterdam, The Netherlands; Division of Molecular Carcinogenesis, NKI Robotics and Screening Center, The Netherlands Cancer Institute, Amsterdam, The Netherlands; Division of Tumor Biology and Immunology, The Netherlands Cancer Institute, Amsterdam, The Netherlands; Division of Tumor Biology and Immunology, The Netherlands Cancer Institute, Amsterdam, The Netherlands

## Abstract

Impaired control of the G1/S checkpoint allows initiation of DNA replication under non-permissive conditions. Unscheduled S-phase entry is associated with DNA replication stress, demanding for other checkpoints or cellular pathways to maintain proliferation. Here, we uncovered a requirement for ADARp150 to sustain proliferation of G1/S-checkpoint-defective cells under growth-restricting conditions. Besides its well-established mRNA editing function in inversely oriented short interspersed nuclear elements (SINEs), we found ADARp150 to exert a critical function in mitosis. ADARp150 depletion resulted in tetraploidization, impeding cell proliferation in mitogen-deprived conditions. Mechanistically we show that ADAR1 depletion induced aberrant expression of Cyclin B3, which was causative for mitotic failure and whole-genome duplication. Finally, we find that also *in vivo* ADAR1-depletion-provoked tetraploidization hampers tumor outgrowth.

## Graphical Abstract

**Graphical Abstract:**
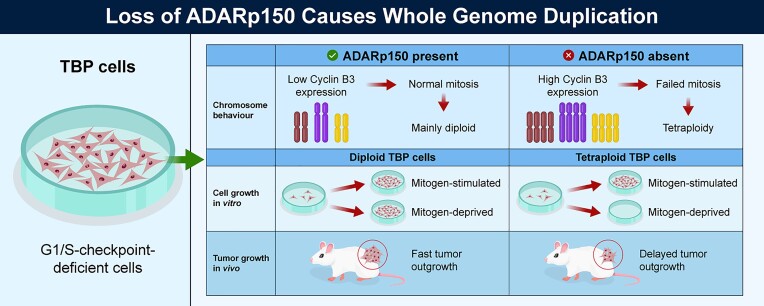

## Introduction

Evading growth suppressors and resisting cell death have been identified as hallmarks of cancer (1). Many cancers have lost the canonical tumor suppressor pRB or other proteins that activate the G1/S checkpoint to prevent DNA synthesis in the absence of mitogenic signaling. This condition is often accompanied by loss of the guardian of the genome p53 that halts cell cycle progression or induces apoptosis in response to overwhelming DNA damage. Further suppression of cell death is frequently achieved by amplification of the anti-apoptotic protein Bcl2. To mimic these hallmarks of cancer, we previously disrupted all three retinoblastoma family members in Mouse Embryonic Fibroblasts (Triple Knock Out (TKO) MEFs), alleviating the G1/S checkpoint. Indeed, these cells can enter S-phase under mitogen-deprived conditions but consequently suffer from severe DNA replication stress, leading to cell cycle arrest and apoptosis. Evading these responses was achieved by overexpression of Bcl2 (TB) and disruption of p53 (TBP). The concomitant suppression of apoptosis and cell cycle arrest allowed G1/S-checkpoint-defective cells to proliferate mitogen independently, thus mimicking in a cell culture model the unrestrained proliferative capacity of cancer cells (2,3). However, mitogen-independently-proliferating TBP cells still suffered from DNA replication stress and critically relied on the intra-S-phase checkpoint proteins ATR and CHK1. In shRNA and CRISPR/Cas9 drop-out screens, we sought to identify additional pathways essential for TBP cells to mitigate the deleterious consequences of replication stress: the loss of specific shRNAs or gRNAs from libraries points to pathways that are needed to sustain proliferation under growth-restricting conditions (4).

Here, we demonstrate that TBP cells critically relied on ADAR1 to maintain mitogen-independent proliferative capacity. ADAR1 has recently received much attention as an RNA editing enzyme that catalyzes the conversion of adenosine (A) to inosine (I) in double-stranded RNA (dRNA). Although the A to I conversion by ADAR1 can recode transcripts, editing by ADAR1 predominantly occurs in non-coding regions such as introns and 3′ untranslated regions (5). *ADAR1* produces two protein isoforms, ADARp110 and ADARp150, containing three dRNA binding domains (dRBD1-3) and a catalytic deaminase domain. Unique to ADARp150 is the Zα-domain that allows binding to left-handed Z-RNA/Z-DNA (6). The short isoform ADARp110 is constitutively expressed in the nucleus and has been implicated in resolving R-loops at telomeres (7). The longer ADARp150 is well known for its role in editing inversely oriented short interspersed nuclear elements (SINEs) found in endogenous mRNAs. Editing of such RNA structures prevents recognition by a family of cytosolic pattern recognition receptors known as retinoic acid-inducible gene I (RIG-I)-like receptors (RLRs), key sensors of virus infection (8). Recent studies suggested activation of this pathway to be causative for developmental defects in patients suffering from the type 1 interferonopathy Aicardi-Goutières syndrome (AGS), who carry an inherited defect in *ADAR1* (6,9).

We uncovered an unexpected role of ADARp150 in proper chromosome segregation during mitosis: upon ADAR1 depletion, TBP cells gradually became tetraploid by occasional whole genome duplication (WGD) events. Tetraploidy turned out to hamper mitogen-independent proliferation of TBP cells, thus explaining the requirement for ADAR1. Mechanistically we show ADARp150 depletion induced overexpression of Cyclin B3 that was critical for WGD. Finally, we show that tetraploidy also hampered tumor outgrowth *in vivo* suggesting that *in vitro* growth-restricting rather than growth-promoting culturing conditions reflect the tumor microenvironment *in vivo*.

## Materials and methods

### Generating cell lines

TBP MEFs were generated and cultured as described previously (4). WT and p53^−/−^ hTERT ARPE-19 cells were kindly provided by Job de Lange (10) (ATCC Cat# CRL-2302, RRID: CVCL_0145). CRISPR/Cas9 technology was used to inactivate *Rb (sgRNA: 5′-TGAACGACATCTCATCT-3′), Rbl1 (sgRNA: 5′-TTTCGTGAACGTATAGAA-3′) and Rbl2 (sgRNA: 5′-CGAGGTTGCTCCTCTTGA-3′)* in these cells. Bcl2 cDNA was overexpressed using retroviral transduction (LZRS-ZEO-Bcl2^3^). These triple knockout, Bcl2 overexpressing and p53^−/−^ hTERT ARPE-19 cells (TBP ARPE) were cultured in the presence of 10% FCS (Capricorn) supplemented with 0.1 mg/ml penicillin-streptomycin (Sigma). *Adar1* knockout clones (TBP MEF) were generated using CRISPR/Cas9 (mouse sgRNA *5′-*TACTCTAACAACCCGCTGAC*-3′*). Stable *ADAR1* and *CCNB3* knockdown TBP ARPE cultures were prepared by lentiviral transduction of pLKO.1 shRNA vectors from the human TRC v1.0 collection (NT: Empty pLKO.1 vector, *Adar1*: shRNA #1 TRCN0000050788 and shRNA #2 TRCN0000050789, *CCNB3*: shRNA #1 TRCN0000006461 and shRNA #2 TRCN0000006462). Concomitant gene knockout clones of *IFNAR1 (sgRNA: 5′-GGCGTGTTTCCAGACTGTTT-3′), IFIH1 (sgRNA: 5′-TTGGACTCGGGAATTCGTGG-3′), RIG-1 (sgRNA: 5′-TCAGGCTGAGAAAAACAACA-3′), Eif2AK2 (sgRNA 5′-GCAACCTACCTCCTATCATG-3′), ZBP1 (sgRNA: 5′-TGGGACACAGCAATGAGATG-3′) and RNASEL (sgRNA: 5′-GCGTGTTTGGATGTGCACAG-3′)* were generated using CRSIPR/Cas9 technology. sgRNAs were designed using CRISPick software (11). FUCCI constructs CSII-EF-MCS-mKO-hCdt1 (30/120) and CSII-EF-MCS-mAG-hGem (1/110) were introduced into TBP cells by lentiviral transduction and FACS-sorted to obtain double-positive cultures. ADARp110 and ADARp150 cDNA was reconstituted into TBP cells by transfecting Pvu1-linearized pm-GFP-ADAR-p110 (Addgene #117928), pmGFP-ADAR-p150 (Addgene #117927) or control pmGFP (Addgene #117926) (vector only). Transfected cells were passed in 0.5 mg/ml G418 until non-transfected control cells were cleared. ADAR-p150 mutant cDNA was created by site-directed mutagenesis PCR of the pmGFP-ADAR-p150 plasmid. ADARp150-E912A primers: 5′-ACTGCCATGCAG**C**AATAATCTCCCG-3′ & 5′-CGGGAGATTATT**G**CTGCATGGCAGT-3′. ADARp150-Zα^mut^ primers: 5′-AAATC**T**ATCGAGTTTTA**GC**CTCCCTGGCA-3′ & 5′-TGCCAGGGAG**GC**TAAAACTCGAT**A**GATTT-3′.

### Cell culture

Doubling time of cell cultures was measured by growing 0.15 × 10^6^ cells in a 10 cm culture dish for 3–4 days. Cells were counted and the doubling time was calculated using the following formula: incubation time * ln(2)/ln(final cell count/seeding cell count) (https://www.omnicalculator.com/biology/cell-doubling-time). For serum starvation cell were seeded and allowed to attach for 4 h, washed with PBS, and incubated for the indicated number of days in serum deprived media. To generate growth curves, we grew cells in μClear 96-well plate from Greiner and imaged using IncuCyte ZOOM instrument (Essen Bioscience) every 4 h.

### Flow cytometry

The fraction diploid and tetraploid G1 cells on FACS were analyzed using the FUCCI cell cycle reporters and DAPI. Asynchronous FUCCI^+^ cultures were harvested using a two-step fixation protocol. First for 10 min in 4% formaldehyde and subsequently in 90% ice-cold methanol. Before flow cytometry analysis using the LSR2 SORP (BD Biosciences) cells were stained with 7 mg/ml DAPI. FACS-sorting of diploid and tetraploid TBP ARPE cells was performed by using live-cell fluorescent dye Hoechst 33342 (Invitrogen). To detect cell death among serum starved TBP MEFs we used Zombie NIR^TM^ Fixable Viability Kit (Biolegend). Finally, cell cycle profiling was performed as described previously (2). In short, TBP ARPE cells were labeled with 10 mM BrdU, ethanol-fixed and stained using propidium iodide. All FACS data was analyzed using FloJo^TM^ software version 10.7.1 (Becton Dickson & Company).

### Chromosome spreads

Cells were treated for with 0.1 μg/ml KaryoMAX^TM^ Colcemid^TM^ (Gibco) for 3 hours before harvesting. Next, harvested cells were resuspended in 75 mM KCl solution for 10 minutes and fixed using 3:1 methanol/glacial acetic acid. Fixed cells were dropped onto IHC microscopy slides (DAKO) and stained with 1 mg/ml DAPI. Chromosome spreads were imaged using the metafer system (Metasystems)

### Neutral comet assay

Neutral comet assay was performed according to Olive *et al.* (12) In short, TBP ARPE cells were harvested and embedded in 1% low agarose gel onto CometSlide (R&D Systems), electrophoresed and stained with propidium iodide. Finally, comets were imaged using the inverted Zeiss AcioObserver Z1 microscope (63× objective) and analyzed using CASP software (2).

### DNA fiber assay

DNA fibers were performed as described earlier (2). Briefly, cells were labeled with 25 μM CldU followed by 250 μM IdU, both 20 minutes. Next, cells were harvested, lysed, and spread onto microscopy slides (Dako). After fixation using 3:1 methanol:glacial acetic acid slides were incubated in 2.5 M HCl for 1 h and 15 min. Rat-anti-BrdU (Novus Cat# NB 500-169, RRID: AB_341913, 1:500) and mouse-anti-BrdU (BD Biosciences Cat# 340649, RRID: AB_400443, 1:750) were used to detect CldU and IdU, respectively. After washing, the primary antibodies were fixed using 4% paraformaldehyde for 10 min. Finally, slides were incubated with goat-anti-mouse Alexa-fluor^TM^ 488 (Thermo Fisher Scientific Cat# A-21131, RRID: AB_2535771, 1;500) and goat anti-rat Alexa-fluor^TM^ 555 (Thermo Fisher Scientific Cat# A-21434 (also A21434), RRID: AB_2535855, 1:500) for 1 h and 30 min and imaged using the 63× objective of the inverted Zeiss AcioObserver Z1 microscope. ImageJ software (ImageJ, RRID: SCR_003070) was used to assess the replication fork speed (1 μm = 2.59 kb (13)).

### Immunoblotting

Protein lysates were prepared using ELB buffer (150 mM NaCl, 50 mM Hepes pH 7.5, 5 mM EDTA, 0.1% NP-40) supplemented with protease inhibitors (Roche). Protein concentration was determined using the BCA protein assay kit (Roche), as described previously (10). 20 μg protein was loaded onto 4–12% NuPAGE Bis–Tris gels (Life Technologies). Primary antibodies used: ADAR1 (Santa Cruz Biotechnology Cat# sc-73408, RRID: AB_2222767), RB1 (Santa Cruz Biotechnology Cat# sc-50-G, RRID:AB_632340), RBL1 (Santa Cruz Biotechnology Cat# sc-318, RRID: AB_2175428), RBL2 (D09855-2 Oncogene), BCL2 (Santa Cruz Biotechnology Cat# sc-509, RRID: AB_626733), p53 (BD Biosciences Cat# 554293, RRID: AB_395348), IFNAR1 (Thermo Fisher Scientific Cat# PA5-79441, RRID: AB_2746557), MDA5 (Cell Signaling Technology Cat# 5321 (also 5321S), RRID: AB_10694490), RIG1 (D1466 Cell Signaling), PKR (Santa Cruz Biotechnology Cat# sc-6282, RRID: AB_628150), RNAseL (Cell Signaling Technology Cat# 27281, RRID:AB_2798941), ZBP1 (Novus Cat# NBP1-76854, RRID: AB_11018813). Primary antibodies that, in our study, did *not* detect the antibody of interest: ADAR1 (Santa Cruz Biotechnology Cat# sc-271854, RRID: AB_10708553), IFNAR1 (Santa Cruz Biotechnology Cat# sc-7391, RRID: AB_2122749, Thermo Fisher Scientific Cat# MA5-43696, RRID: AB_2912628), RIG1 (Santa Cruz Biotechnology Cat# sc-376845, RRID: AB_2732794), ZBP1 (Cell Signaling Technology Cat# 60968, RRID: AB_2799599, AdipoGen Cat# AG-20B-0010 (also AG-20B-0010-C100), RRID: AB_2490191). IR Dye 800CW secondary igG antibodies (LI-COR) were used. Blots were imaged using the Odyssey imaging system and analyzed using ImageStudioLite software (LI-COR).

### RNA sequencing

300 000 cells were serum starved for 4 days or grown in the presence of FCS. Cells were washed 3 times with PBS and dissociated using trypsin. Collected cells were washed again and 1 × 10^6^–1.5 × 10^6^ were directly resuspended in 800 μl RLT buffer (Roche). Library preparation was performed with the TruSeq polyA stranded RNA prep kit (Illumina) according to the manufacturer protocol. The libraries were analyzed for size and quantity of cDNAs on a 2100 Bioanalyzer using a DNA 7500 chip (Agilent), diluted, and pooled in multiplex sequencing pools. The libraries were sequenced as 51 bp paired-end on a Novaseq 6000 (Illumina) with 20 million reads. Differential expression analysis of the RNAseq data was done with DESeq2 in the statistical programming language R (version 4.0.1) (14). Further downstream exploration and analyses was done in QIAGEN IPA (15).

### RT-PCR

RNA was isolated using the RNAeasy micro kit (QIAGEN). First strand cDNA synthesis was done using SuperScript™ (Thermo Scientific) reverse transcriptase and binding of random hexamer primers. qPCR was performed with 2 ul of 1/5 diluted cDNA using primers specific for *CCNB3* and *GAPDH* using the Lightcycler 480 SYBR green master mix (Roche). Relative expression of *CCNB3* was calculated for each primer combination using *GAPDH* expression of each sample as a loading control for the total cDNA. qPCR primers used for amplification of *CCNB3*: Fw-1 5′-CACACCAACATGAAGACACTGACC-3′, Rv-1 5′-CACAGCCTTGAGACTATCGTAAGAAC-3′, Fw-2 5′-TGGGCAAGTCCAGGACCAC-3′, Rv-2 5′-GGGTTGAAACTTGGATCACTGCT-3′, Fw-3 5′-AGGAACACACATGCTCTTGGACT-3′, Rv-3 5′-TGGTACCACGGTAGTAGAGGCTA-3′. qPCR primers used for *GAPDH* amplification: Fw 5′-ACAACTTTGGTATCGTGGAAGG-3′, Rv 5′-GCCATCACGCCACAGTTTC-3′.

### Time-lapse microscopy

Time-lapse microscopy of the FUCCI fluorophores was performed as described before (2). Images were prepared using the 4 × 4 binning mode. Data was stitched including tile-fusion using 8% overlap and 3% shift. Analysis of individual mitoses was done by manually following single cells using the Zeiss software.

### *Ex vivo* analysis of tumors

NMRI mice (https://janvier-labs.com/en/fiche_produit/nmri_mouse/) were used for xenograft experiments. 2 × 10^6^ FUCCI-expressing TBP ARPE cells, either diploid or tetraploid and either with ADAR1 shRNA or NT shRNA, in 200 μl PBS were injected in a single flank. These experiments were carried out after approval of the animal welfare committee of the Netherlands Cancer Institute. Tumor size was measured bi-weekly with a caliper, tumor volume was calculated (length × width × width/2). Most mice (12/20) were sacrificed when the maximal tolerated volume of 1.5 cm^3^ was reached. The remaining (8/20) mice were sacrificed earlier because of ulcerating tumors (4/20), secondary tumors (2/20) or rectal prolapse (2/20). All these mice formed visible tumors which were analyzed *ex vivo* for the presence of tetraploid cells. We manually minced part of the tumor with a razor (Personna™) and enzymatically in DMEM medium with 3 mg/ml collagenase A (Roche), trypsin and 25 μg/ml DNAse I (Sigma) for 30 min at 37 °C (16). Digested tumor cells were filtered (100 μm) and cultured in DMEM media supplemented with 10% FCS (Capricorn) and 0.1 mg/ml penicillin-streptomycin (Sigma) for 2–4 days depending on the confluency of individual tumors and subsequently subjected to FUCCI FACS experiments to detect the level of tetraploid cells among mKO2-hCDT1^+^ mAG1-hGem^−^ cells.

### Statistical analysis

GraphPad Prism software was used for statistical analysis and the generation of graphs. For survival analysis of animal studies, we used Log-rank tests (Mantel–Cox). One- and two-way ANOVA were used for comparisons of multiple groups. All statistical tests were performed two-tailed and adjusted for multiple testing when appropriate. Sample sizes and specific statistical test used were presented in the legends.

## Results

### ADAR1 is critical for mitogen-independent proliferation

**Figure 1.**
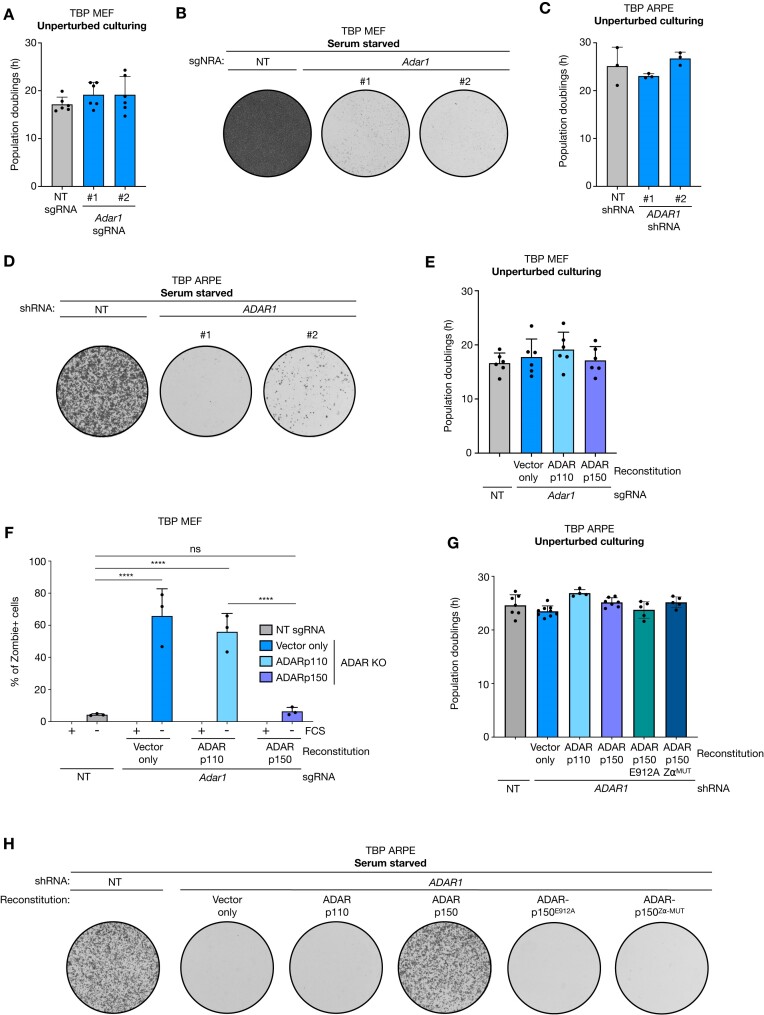
ADAR1 depletion is lethal in non-permissive culturing conditions. (**A**) Population doublings (h) of TBP MEFs transduced with non-targeting (NT) or *Adar1* sgRNAs clones grown in unperturbed culturing conditions (+10% FCS) and passed before reaching confluency. Dots represent 6 independent measurements. Error bars indicate standard deviation. (**B**) Representative image of 300.000 serum-starved TBP MEFs from (A) cells cultured for 10 days in 6-well plates. (**C**) Population doublings (h) of TBP ARPE cells transduced with a NT or *ADAR1* shRNAs in unperturbed culturing conditions (+10% FCS) and passed before reaching confluency. Dots represent 3 independent measurements. Error bars indicate standard deviation. (**D**) Representative image of 25.000 serum-starved TBP ARPEs from (C) grown for 14 days in 6-well plates. (**E**) Population doublings (h) of NT or ADAR1 knockout TBP MEF reconstituted with linearized pmGFP (vector only), pmGFP-ADAR-p110 or pmGFP-ADAR-p150. Cells were grown in unperturbed culturing conditions (+10% FCS) and passed before reaching confluency. Dots represent six independent measurements. Error bars indicate standard deviation. (**F**) Fraction of Zombie^+^ TBP MEFs from (**E**) cultured in unperturbed (+10% FCS) or serum-starved culturing conditions for 7 days. Dots represent three individual experiments. Error bars indicate standard deviation. Asterisks represent adjusted *P*-value of two-sided two-way ANOVA (Šidák multiple comparison test) (****P-value < 0.0001). ns = non-significant. (**G**) Population doublings (h) of NT or ADAR knockdown TBP ARPE cells reconstituted with linearized pmGFP (vector only), pmGFP-ADAR-p110, pmGFP-ADAR-p150, pmGFP-ADAR-p150-E912A or pmGFP-ADAR-p150-Zα^mut^ grown in unperturbed culturing conditions (+10% FCS) and passed before reaching confluency. Dots represent 4–7 independent measurements. Error bars indicate standard deviation. (**H**) Representative images of 25.000 serum starved TBP ARPE cells from (G) cultured for 14 days in 6-well plates.

To identify pathways that are critical for mitogen-independent proliferation of G1/S-checkpoint-defective cells, we performed a genome-wide CRISPR-Cas9 dropout screen. One of the hits of this screen (this unpublished CRISPR screen will be published elsewhere by Van Gemert *et al.*) indicated that TBP MEFs critically relied on ADAR1 for proliferation in non-permissive culturing conditions. We created ADAR1 knockout clones and observed minimal or no effect on the doubling time in unperturbed culturing conditions (Figure 1A and Supplementary Figure S1A). In contrast, upon serum starvation, TBP MEFs critically depended on ADAR1 to maintain proliferative capacity (Figure 1B). Next to the murine TBP MEFs we also created diploid human retinal epithelial cells (ARPE) with the same genetic background. Like TBP MEFs, TBP ARPE cells (Supplementary Figure S1B-D) were able to proliferate in the absence of mitogens and proliferation depended on the intra-S-phase-checkpoint kinase CHK1 (Supplementary Figure S1E). Importantly, also for TBP ARPE cells, ADAR1 depletion (Supplementary Figure S1F) was lethal only in non-permissive culturing conditions (Figure 1C, D).

### ADAR1 depletion does not aggravate DNA replication stress

We previously showed that mitogen-deprived TBP MEFs had reduced replication fork speed (2,10). Since ADARp110 has been implicated in RNA editing of DNA:RNA hybrids at telomeres we wondered whether ADAR1 depletion aggravates DNA replication stress (7,17). Therefore, we studied replication dynamics using the DNA fiber assay. As expected, TBP ARPE cells transduced with a control (non-targeting; NT) shRNA showed reduced replication fork speed and a trend towards reduced levels of origin firing upon serum deprivation, both indicative of replicative stress (Supplementary Figure S1G, H). ADAR1 depleted TBP ARPE cells showed similar changes in replication fork dynamics. In addition, DNA double-stranded break (DSB) formation, measured by the comet assay, was similar in ADAR1-proficient and -depleted cells in non-permissive culturing conditions (Supplementary Figure S1I). These results suggest that ADAR1 depletion is synthetic lethal in serum-starved conditions for reasons other than aggravation of DNA replication stress.

### ADARp150 is essential in mitogen-deprived conditions

The knockout/knockdown of ADAR1 affected both the short (ADARp110) and long isoform (ADARp150) of ADAR1. To study the effects of the two isoforms separately, we performed reconstitution experiments using ADARp110 and ADARp150 cDNA. In both, mouse and human TBP cells, we observed rescue of mitogen-independent proliferation upon reconstitution by ADARp150 but not by ADARp110 (TBP MEFs Figure 1E-F and TBP ARPE in Figure G-H). Both isoforms were found to have extensive overlapping editing sites, as expected given their shared dRBD and deaminase domain (18). The Zα domain, however, is unique to the ADAR-p150 isoform and allows binding to a left-handed structure of RNA and DNA called Z-RNA/Z-DNA (6). We created point mutations in the ADARp150 cDNA expression plasmid to inactivate the Zα-domain (N173A/Y177A) or catalytic deaminase-domain (E912A) and expressed these ADARp150 mutants in ADAR1-depleted cells (6,7). In contrast to wild-type (WT) ADARp150 cDNA, reconstitution with these mutants failed to rescue mitogen-independent proliferation (Figure 1H), suggesting that both the Zα- and deaminase domain of ADARp150 are critical in supporting mitogen-independent proliferation.

### ADAR1 depletion does not affect interferon signaling of TBP cells

The consequences of ADAR1 deficiency have been studied in mice modelling the type 1 interferonopathy Aicardi-Goutières syndrome (AGS). Over 60% of AGS cases are caused by an inherited point mutation (p.P193A) in the Zα domain of ADAR1 combined with a defective deaminase domain or *ADAR1*-null allele (19,20). In mice, ADAR1 deficiency triggered the integrated stress response (ISR) causing postnatal mortality. This could be rescued by an ISR inhibitor (ISRIB), or knockout of components of this pathway (9).

We therefore considered the possibility that the ISR was responsible for the massive death of serum-starved ADAR1-deficient TBP ARPE cells. Surprisingly though, in our hands, knockouts of the type 1 IFN receptor (*IFNAR1*), the RIG1-like receptor (RLR) family members MDA5 (*IFIH1*), LGP2 (*DHX58*), RIG-1 (*RIG-1*), PKR (*EIF2AK2*) or endoribonuclease RNAseL (*RNASEL*) (Supplementary Figure S2A-G and I) did not rescue mitogen-independent proliferation of ADAR1-depleted TBP cells, nor did ISRIB (Supplementary Figure S2I). Similarly, disruption of the Z-DNA/Z-RNA binding protein 1 (ZBP1), another mediator of postnatal lethality in AGS mouse models (21–24), did not rescue lethality in serum-starved, ADAR1 depleted TBP ARPE cells (Supplementary Figure S2A, H and I).

Finally, we used total RNA sequencing to assess transcripts and pathways that were differentially expressed upon ADAR1 depletion and ADARp150 reconstitution (Supplementary Figure S2J). Unexpectedly, in mitogen-proficient conditions, ADAR1 depletion reduced interferon signaling. Introduction of ADARp150 rescued interferon signaling and stimulated pathways involving RIG1-like receptors and PKR. Reduced interferon signaling upon ADAR1 depletion and rescue by ADARp150, were also seen in the absence of mitogens, albeit both to a lesser extent and accompanied by a slight dampening of the RIG1-like receptors pathway as well as the NF-κB activation and JAK/STAT signalling. Collectively, these analyses make it unlikely that suppression of type 1 interferon signalling explains the requirement for ADARp150 for mitogen-independent proliferation.

### ADAR1 depletion results in tetraploidy

**Figure 2.**
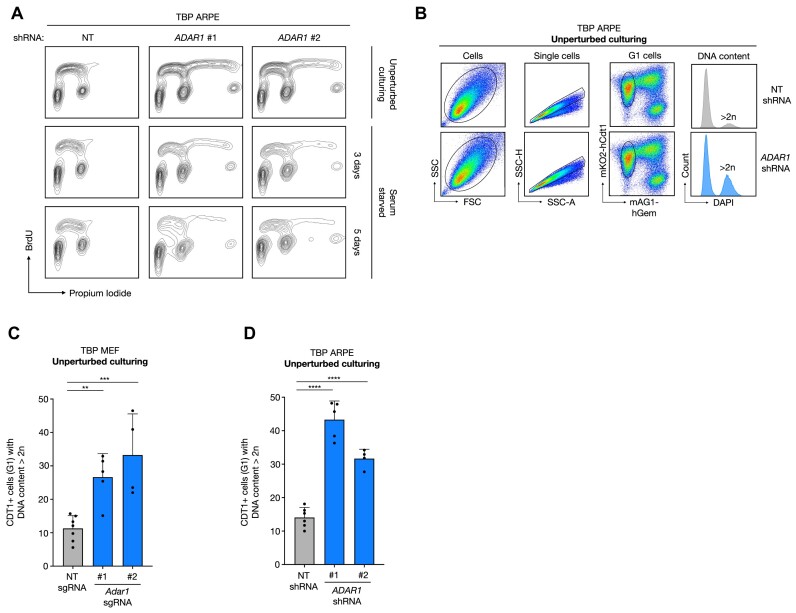
ADAR1 depletion induces tetraploidization of TBP cells. (**A**) Cell cycle profiling of BrdU pulse-labeled TBP ARPE cells transduced with a NT or *ADAR1* shRNA grown in the presence or absence of serum for the indicated days. Propidium iodide was used to detect DNA content. (**B**) Gating strategy of FUCCI FACS protocol (26) used to quantify the fraction of G1 cells with a DNA content larger than 2n. Cells (1^st^ column) were selected based on side-scatter (SSC) and forward scatter (FSC), single cells were separated by side-scatter-area (SSC-A) and side-scatter-height (SSC-H), cells gated by using mKO2-hCDT1^+^/mAG1-hGem^−^ cells represent G1 cells and finally DAPI was used to identify G1 cells with a DNA content larger than 2*n*. (C, D) Percentage of G1 cells with a DNA content larger than 2n in TBP MEF (**C**) and TBP ARPE (**D**) cultures transduced with control (NT) (grey) and *Adar1* (blue) sgRNA or *ADAR1* shRNA, respectively) cultures. Dots represent independent measurements. Error bars indicate the standard deviation. Asterisks represent adjusted *P*-value of two-sided one way ANOVA test (Dunnett's multiple comparisons test) (**P-value = 0.008, ***P-value = 0.0009, ****P-value < 0.0001).

The absence of increased IFN signaling upon ADAR1 knockdown prompted us to further characterize ADAR1-depleted TBP cells. Pulse labeling of TBP ARPE cells with the thymidine analogue BrdU revealed that ADAR1-depleted TBP ARPE cells in both, perturbed and unperturbed conditions, contained a significant fraction of cells with tetraploid DNA content (Figure 2A). To distinguish diploid cells in G2- and tetraploid cells in G1-phase (both 4n and BrdU^Neg^), we used FUCCI cell cycle reporters (25) that allowed us to measure the DNA content of G1 cells in asynchronous cultures (Figure 2B) (26). In both, ADAR1-depleted TBP ARPE and TBP MEFs cultured in the presence of mitogens, G1 cells contained significantly more cells with a DNA content larger than 2*n* (Figure 2C, D).

As we are not aware of studies describing a role for ADAR1 in preventing whole genome duplication (WGD), we wondered whether tetraploidization was specific for TBP cells. p53 depletion alone already significantly increased the fraction of cells with a DNA content larger than 2*n* in ARPE cells and, to a lesser extent, also in HCT116 cells. ADAR1 depletion aggravated tetraploidization in both cell types, but not in the corresponding ADAR1-proficient cells (Supplementary Figure S3A–D). On the other hand, we did not observe tetraploidization in p53KO-MCF7 cells with or without ADAR1 depletion (Supplementary Figure S3E-F). These experiments indicate that p53 serves as a powerful safeguard against spontaneous and ADAR1-depletion-induced whole genome duplication, although other (cell-type-specific) protection mechanism likely operate as well.

### ADARp150 depletion promotes WGD

**Figure 3.**
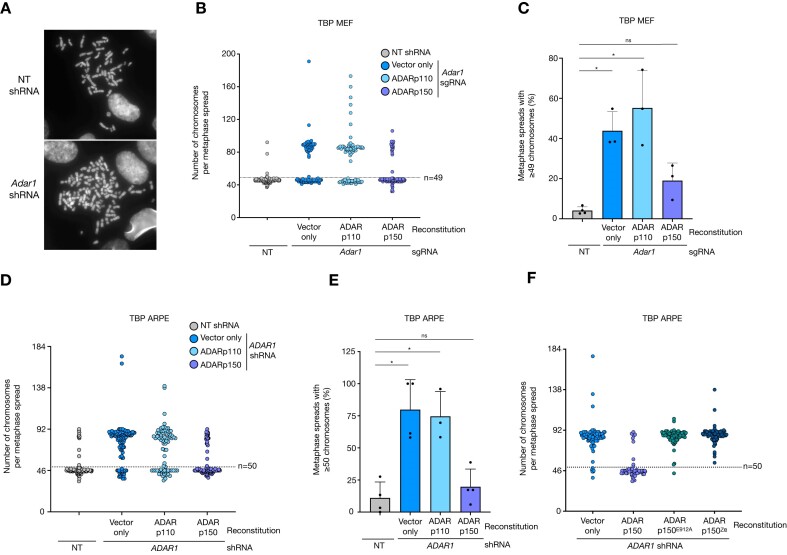
ADARp150 depletion causes whole genome duplication. (**A**) Examples of individual chromosome spreads of non-targeting (NT) and *ADAR1* shRNA transduced TBP ARPE cells. (**B**) Dots represent the number of chromosomes in individual chromosome spreads of NT (grey) and ADAR1 knockout TBP MEF clone reconstituted with linearized pmGFP (vector only; blue), pmGFP-ADAR-p110 (light blue) and pmGFP-ADAR-p150 (purple). Cells were grown in unperturbed culturing conditions (+10% FCS). Dashed horizontal line represent a cut-off of *n* = 49 below which cells are considered (near-)diploid. (**C**) Quantification of the fraction of cells with a chromosome count larger or equal to 49 in NT (grey) and ADAR1 knockout TBP MEF cultures reconstituted with the indicated vectors. Dots represent independent experiments which were combined in (B). Error bars indicate the standard deviation. Asterisk show statistical adjusted *P*-value by one-way ANOVA (Kruskal–Wallis test). Comparisons to NT sgRNA: vector only *P*-value = 0.0295, ADARp110 *P*-value = 0.0151, ADARp150 *P*-value = 0.716. (**D**) Number of chromosomes in individual chromosome spreads of TBP ARPE cells transduced with a NT (grey) and *ADAR1* shRNA, reconstituted with linearized pmGFP (vector only; blue), pmGFP-ADAR-p110 (light blue), pmGFP-ADAR-p150 (purple). Cells were grown in unperturbed culturing conditions (+10% FCS). Dashed horizontal line represent a cut-off of *n* = 50 below which cells are considered diploid. (**E**) Quantification of the fraction of cells with a chromosome count larger or equal to 50 in TBP ARPE cultures transduced with a NT (grey) or *ADAR1* shRNA and reconstituted with the indicated vectors. Dots represent independent experiments which were combined in (D). Error bars indicate the standard deviation. Asterisk show adjusted *P*-value by one-way ANOVA (Kruskal–Wallis test). Comparisons to NT shRNA: vector only *P*-value = 0.0167, ADARp110 *P*-value = 0.0498, ADARp150 *P*-value > 0.999. (**F**) Number of chromosomes in individual chromosome spreads of TBP ARPE cells transduced with an *ADAR1* shRNA reconstituted with linearized pmGFP (vector only; blue), pmGFP-ADAR-p150 (purple), pmGFP-ADAR-p150-E912A (dark-green) and pmGFP-ADAR-p150-Zα^mut^ (dark-blue). Cells were grown in unperturbed culturing conditions (+10% FCS). Dots represent the number of counted chromosomes in individual chromosome spreads.

We next tested whether reconstitution of ADAR1-depleted cells with wild-type or mutant ADAR1 would affect the formation of tetraploid cells. To this end, we prepared chromosome spreads of all ADAR1-reconstituted cell lines and counted the number of chromosomes (Figure 3A). Diploid TBP MEFs (mouse origin) are expected to have 40 chromosomes while TBP ARPE (human origin) should contain 46 chromosomes per cell. Although for both cell lines we measured considerable heterogeneity in chromosome counts, most TBP cells had a near-diploid chromosome count (Figure 3B and D; below horizontal dashed line). However, among ADAR1-depleted TBP MEFs and ARPE cells we found a sizable population with approximately twice the expected chromosome content (Figure 3B and D; vector only). ADARp150 reconstitution clearly reduced the number of cells with an abnormal karyotype in both TBP cell types (Figure 3B, D; quantified in Figure 3C, E), while ADARp110 had no effect. This effect of ADARp150 required its catalytic and Z-RNA/Z-DNA-binding activity as ADAR1-depleted TBP ARPE cells reconstituted with catalytic dead or Zα-domain-defective mutant cDNA remained highly tetraploid (Figure 3F). Finally, in ADAR1-depleted *ifnar1^−/−^* TBP ARPE cells, which were unable to proliferate in mitogen-independently, we detected high levels tetraploidy, indicating suppression of whole genome duplication by ADARp150 was not mediated by suppression of the ISR (Supplementary Figure S3G). These results show that ADARp150 depletion not only restricted mitogen-independent proliferation but also induced tetraploidization of TBP cells.

### ADAR1 depletion blocks mitogen-independent proliferation of tetraploid cells

**Figure 4.**
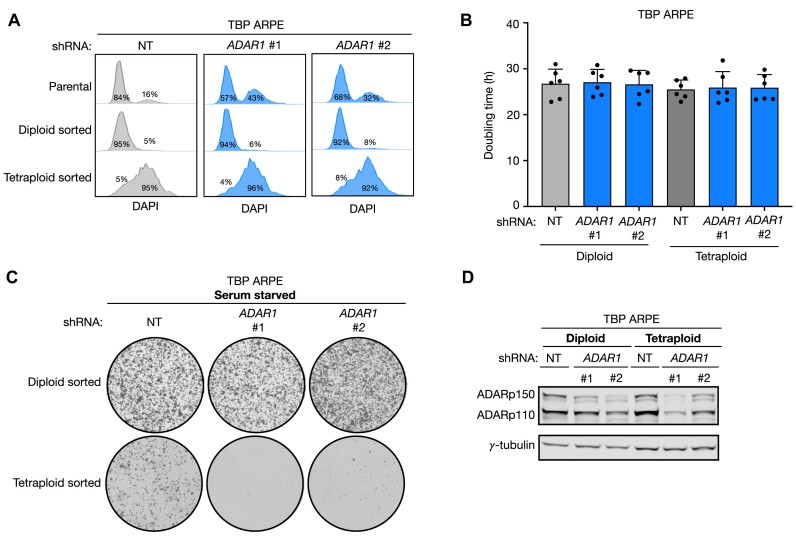
Progressive polyploidization upon ADAR1 depletion causes lethality under mitogen-deprived conditions. (**A**) DNA content (DAPI) of G1 TBP ARPE cells transduced with non-targeting (NT; grey) or *ADAR1* shRNA (blue). The 1st row represents the parental population. The 2nd and 3rd row represent the diploid- and tetraploid-sorted fraction, respectively, that were FACS-sorted from the parental cultures. (**B**) Population doublings (h) of diploid- (left three bars) and tetraploid-sorted (right three bars) TBP ARPE cells transduced with NT (grey) and *ADAR1* shRNA (blue). Cells were grown in unperturbed culturing conditions (+10% FCS) and passed before reaching confluency. Dots represent six independent measurements. Error bars indicate standard deviation. (**C**) Representative images of 25 000 diploid- and tetraploid-sorted TBP ARPE cells from (**B**) after 14 days of serum starved culturing. Plates were stained with crystal violet. (**D**) Protein levels of ADARp110 and ADARp150 of cultures used in (B) and (C). γ-Tubulin is used as a loading control.

Does genome duplication upon ADARp150 loss underly the synthetic lethal interaction we observed between ADAR1 depletion and serum starvation? To test the effect of WGD on mitogen-independent proliferation, we used the flow cytometry-based method described above (Figure 2B) to sort diploid and tetraploid G1 TBP cells with or without ADAR1 depletion (Figure 4A). FACS-sorted populations were maintained in unperturbed culturing conditions and showed similar doubling times (Figure 4B). However, in serum-starved conditions tetraploidy reduced the proliferative ability of control TBP ARPE cells (Figure 4C; compare NT shRNA diploid- and tetraploid-sorted). Furthermore, while during the short 2-week culturing period used here ADAR1 knockdown (Figure 4D) did not grossly affect the proliferation of sorted diploid TBP cells, ADAR1-depleted tetraploid TBP cells were unable to proliferate upon mitogen deprivation (Figure 4C). These results indicate that mitogen-independent proliferation is negatively affected by tetraploidy and even completely blocked upon continuing polyploidization in the absence of ADAR1.

### Mitotic failures in the absence of ADAR1 activity underlies WGD

**Figure 5.**
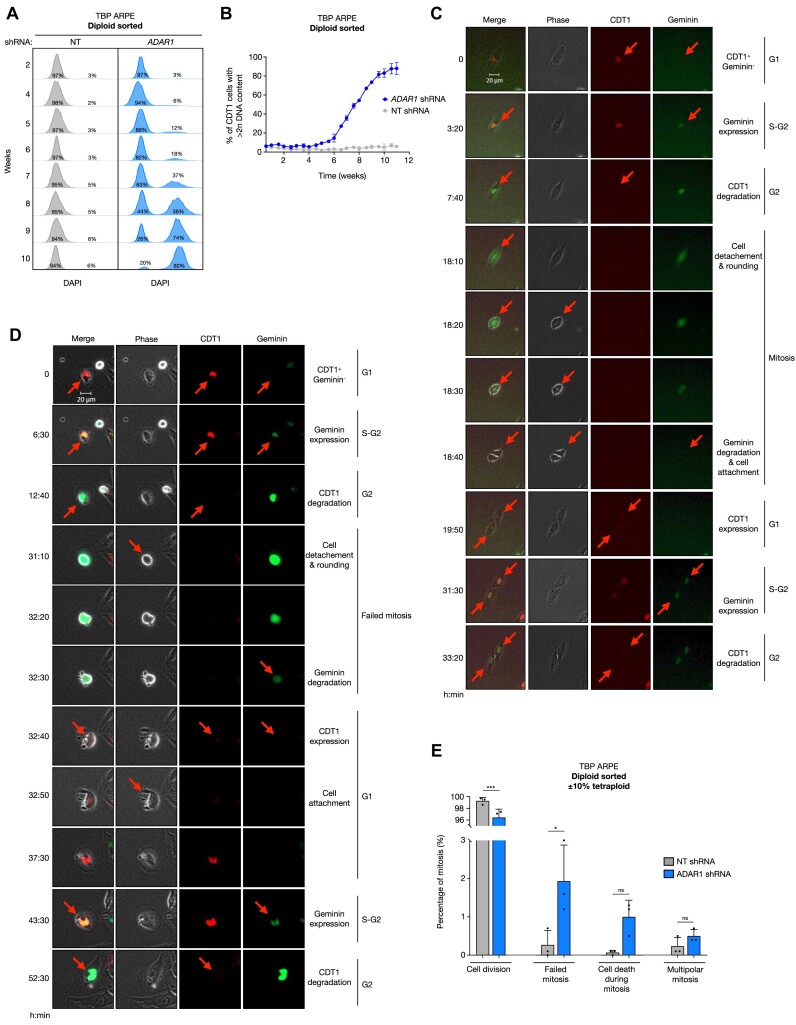
Mitotic failures in the absence of ADAR1 activity underlies WGD. (**A**) DNA content (DAPI) of diploid sorted G1 TBP ARPE cells transduced with a non-targeting (NT; grey) and *ADAR1* shRNA (blue), cultured over time (rows; weeks). Percentages represent the fraction of G1 cells with 2n (left) or > 2n (right) DNA content. (**B**) Percentage of TBP ARPE cells in G1 phase transduced with a non-targeting (NT; grey) and *ADAR1* shRNA (blue) with a DNA content > 2*n* cultured over time (weeks) based on the method described in Figure 2C. Cultures were measured on FACS bi-weekly and passed in unperturbed culturing conditions (+10% FCS). (**C**) Representative example of a FUCCI expressing TBP ARPE cell transduced with a NT shRNA tracked through mitosis using time-lapse microscopy (20× objective, 10-min intervals). Pictures represent key events (arrows and text) over time (rows; hours:minutes). (**D**) Representative example of a FUCCI expressing TBP ARPE cell transduced with a shRNA targeting *ADAR1* that underwent a mitotic failure, tracked using time-lapse microscopy (20× objective, 10-min intervals). Pictures represent key events (arrows and text) over time (rows; hours:minutes). (**E**) Percentage of successful cell divisions and failed mitotic events as a percentage of all mitosis. Bars indicate TBP ARPE cells transduced with a NT (grey) or *ADAR1* shRNA (blue). Sample size: NT shRNA (*n* = 1704) and *ADAR1* shRNAs (*n* = 1519) individual mitosis divided over three independent experiments (dots). Error bars indicate the standard deviation. Asterisks represent adjusted p-value of a two-sided one-way ANOVA test (Šidák multiple comparison test). Significance levels per category: ‘Cell division’ ***P-value = 0.0005, ‘Failed mitosis’ *P-value < 0.0113, ‘Cell death during mitosis’ ns = non-significant; *P*-value = 0.2020.

The tetraploid cells we observed in the absence of ADARp150 likely resulted from WGD events. To better understand the onset of tetraploidy we cultured diploid-sorted TBP ARPE cells, control and ADAR1 knockdown, over time in unperturbed conditions. It appeared that tetraploidy developed gradually, visibly starting to accumulate after 5–6 weeks, and reaching near-completion in approximately 10 weeks (Figure 5A, B). To visualize WGD events, we followed FUCCI-expressing TBP ARPE cells over time by time-lapse microscopy. The alternation of FUCCI fluorophores in combination with physical separation of individual cells revealed normal mitotic progression and successful cell division in the majority of both control and ADAR1 depleted cells (Figure 5C, Supplementary Video S1). However, upon ADAR1 depletion we observed increased levels of failed cell divisions, in up to 2% of all mitoses. Consistent with the accumulation of tetraploid cells, these TBP ARPE cells generally continued the cell cycle and remained viable until the end of the experiment (Figure 5D, E and Supplementary Video S2–S6). These data suggest that the accumulation of tetraploid karyotypes upon ADAR1 depletion likely resulted from a mitotic defect.

### Tetraploidization upon ADARp150 depletion by induction of Cyclin B3

**Figure 6.**
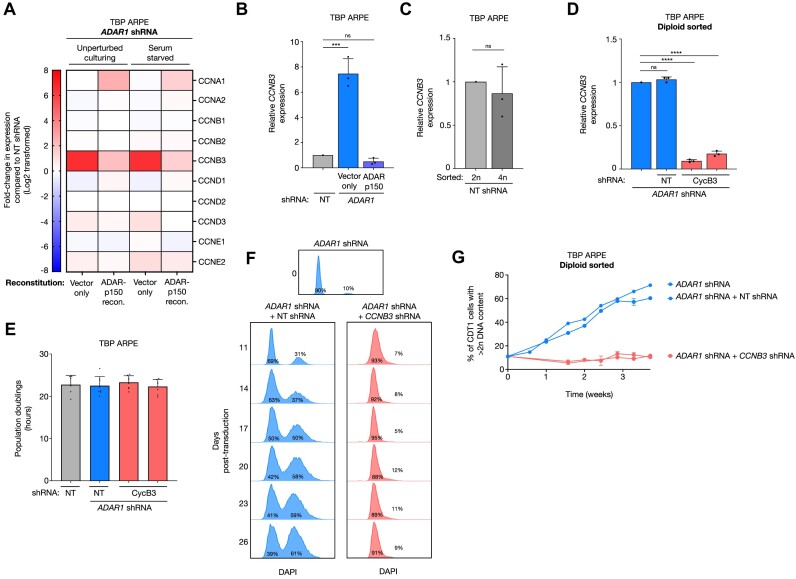
Tetraploidization in TBP ARPE cells upon ADARp150 depletion occurs through induction of Cyclin B3. (**A**) Expression levels of the major cyclins in TBP ARPE cells transduced with an *ADAR1* shRNA reconstituted with pmGFP (vector only) or pmGFP-ADARp150 compared to TBP ARPE cells transduced with a NT shRNA, cultured as indicated. Data was obtained by total RNA sequencing. Color indicates the log_2_-fold change. (**B**) Relative *CCNB3* expression levels of indicated TBP ARPE cells obtained using RT-PCR. Dots represent independent primer pairs specific for *CCNB3* cDNA. Asterisks represent adjusted p-value of a two-sided one-way ANOVA test (Šidák multiple comparison test) (***P-value 0.0001) (**C**) Relative *CCNB3* expression levels of indicated TBP ARPE cells obtained using RT-PCR. Dots represent independent primer pairs specific for *CCNB3* cDNA. (**D**) Relative *CCNB3* expression levels of indicated TBP ARPE cells obtained using RT-PCR. Dots represent independent primer pairs specific for *CCNB3* cDNA. Asterisks represent adjusted p-value of a two-sided one-way ANOVA test (Šidák multiple comparison test) (****P-value < 0.0001). (**E**) Population doublings (h) of indicated TBP ARPE cells grown in unperturbed culturing conditions (+10% FCS) and passed before reaching confluency. Dots represent independent measurements. Error bars indicate standard deviation. (**F**) Upper plot represents the DNA content (DAPI) of diploid sorted G1 TBP ARPE cells transduced with an *ADAR1* shRNA that accumulated 10% tetraploidy. These cells were used to transduce a non-targeting (NT) shRNA (blue; left lower column) or *CCNB3* shRNA (red; right lower column) cultured over time (rows; days post-transduction). Percentages represent the fraction of G1 cells with 2*n* (left) or >2*n* (right) DNA content. (**G**) Percentage of TBP ARPE cells in G1 phase transduced with an *ADAR1* shRNA (blue), *ADAR1* shRNA + non-targeting (NT) shRNA (blue) or an *ADAR1* shRNA + *CCNB3* shRNA (red) with a DNA content >2*n* cultured over time (weeks) based on the method described in Figure 2C. Cultures were measured on FACS bi-weekly and passed in unperturbed culturing conditions (+10% FCS).

The editome of ADAR1 includes a wide range of transcripts and pathways whose expression might be affected by A to I editing (27,28). Notably, ADAR1 has been shown to promote expression of transcripts associated with DNA replication and meiotic synapsis (29). Ingenuity pathway analysis (IPA) of our RNA sequencing data set (Supplementary Figure S2J) did not reveal notable changes in selected pathways related to cell cycle control, DNA replication and mitosis upon ADAR1 depletion (Supplementary Figure S4A). However, within the ‘mitotic roles of polo-like kinase’ pathway we observed a strong (±95-fold) induction of *CCNB3* in ADAR1-depleted cells. *CCNB3* encodes Cyclin B3, the third member of the B-type cyclin family. B-type cyclins are induced at the end of G2 phase and by activating cyclin-dependent-kinase-1 (CDK1) drive mitotic initiation and progression (30–32). Cyclin B3-CDK1 has been shown to activate the anaphase-promoting complex/cyclosome (APC/C) to promote metaphase to anaphase transition (33–35). Moreover, overexpression of non-degradable Cyclin B3 halts mitotic progression in late anaphase (36). Aberrant expression of *CCNB3* during mitosis could thus underlie the WGD events that we observed upon ADAR1 depletion. First, we confirmed that in our RNA sequencing dataset, despite some minor variations, *CCNB3* was the only cyclin whose expression was induced upon ADAR1 depletion and restored upon ADARp150 reconstitution (Figure 6A). Next, RT-PCR revealed an 8-fold increase of *CCNB3* transcripts upon ADAR1 depletion, which was abolished upon ADARp150 reconstitution, confirming that upregulation of *CCNB3* was specific to ADARp150 depletion (Figure 6B). The *CCNB3* levels in diploid- and tetraploid-sorted TBP ARPE cells transduced with a NT shRNA (Figure 6C) did not differ, indicating that ADAR1 depletion rather than tetraploidization underlies the induction of *CCNB3*. Next, we sought to functionally test the involvement of Cyclin B3 in tetraploidization upon ADAR1 depletion. To this end, we used ADAR1-depleted TBP ARPE cell cultures that had accumulated approximately 10% tetraploidy (±5 weeks after sorting; Figure 5A, B) and introduced a NT control shRNA and two independent shRNAs targeting *CCNB3* (Figure 6D). Having successfully prevented the induction of *CCNB3* upon ADAR1 depletion we used the approach used in Figure 5A-B to monitor the accumulation of tetraploid TBP cells over time. It is noteworthy that depletion of Cyclin B3 in ADAR1-depleted cells did not affect the population doubling time of these cells (Figure 6E). Consistent with previous results, ADAR1-depleted TBP ARPE cultures continued to accumulate tetraploid cells. However, preventing *CCNB3* upregulation by shRNA suppressed the accumulation of tetraploid karyotypes upon ADAR1 depletion (Figure 6F, G). Collectively, these data indicate that ADARp150 depletion in TBP ARPE cells induced Cyclin B3 expression, resulting in mitotic failures and accumulation of tetraploid karyotypes.

### Relevance of ADAR1 depletion and WGD for tumor growth *in vivo*

**Figure 7.**
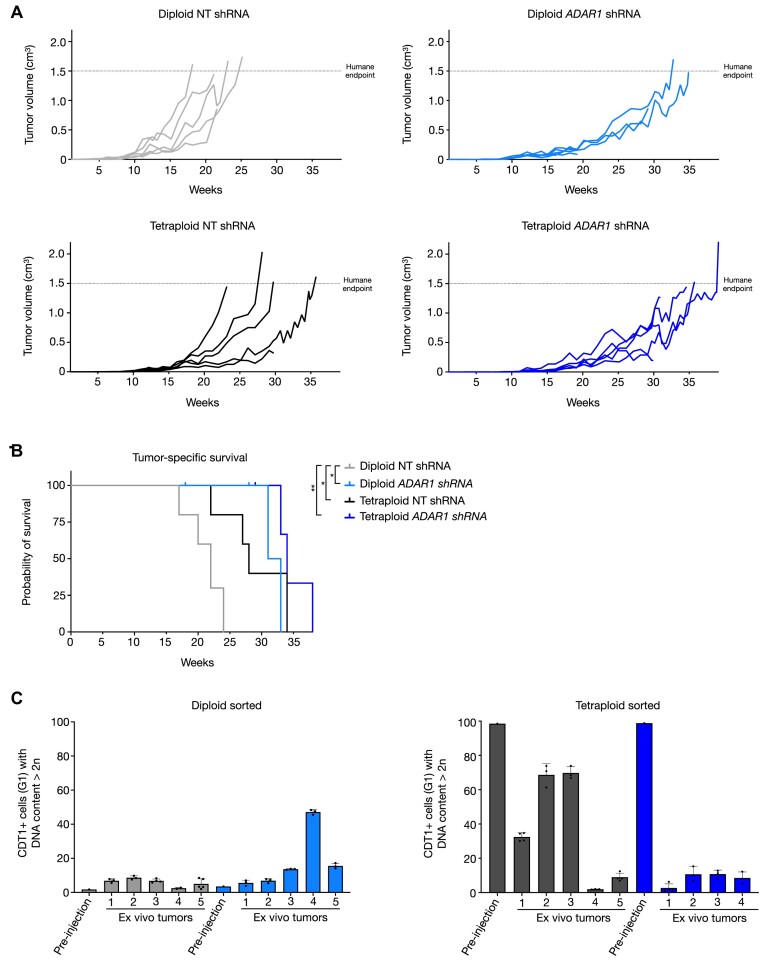
In vivo relevance of ADAR1 depletion and whole genome duplication. (**A**) Individual growth curves of diploid and tetraploid TBP ARPE cells with normal (NT) or reduced ADAR1 expression (*ADAR1* shRNA) injected under the skin of NMRI mice (5 animals per cell line). Dashed line indicates the maximally tolerated tumor size. (**B**) Kaplan–Meier survival curves of mice injected with the indicated cells. Asterisk indicates *P*-value. Significance levels in comparison to Diploid NT shRNA: Diploid *ADAR1* shRNA *P-value = 0.0177, Tetraploid NT shRNA *P-value = 0.0222, Tetraploid *ADAR1* shRNA **P-value 0.0045). (**C**) Percentages of diploid- or tetraploid-sorted TBP ARPE cells with a DNA content larger than 2*n* before injection (pre-injection) and after tumor growth *in vivo* and brief culturing *ex vivo* (*ex vivo* tumors). Grey bars indicate cells with normal ADAR1 expression; blue bars cells with ADAR1 knockdown.

During tumor evolution, the (transient) lack of blood supply to provide sufficient nutrients and oxygen may reflect the non-permissive culturing conditions that we used *in vitro*. Likewise, the microenvironment of G1/S-checkpoint-defective incipient tumor cells may cause unscheduled S-phase entry and DNA replication stress. In this scenario, tetraploidy and ADAR1 depletion would hamper tumor growth of TBP cells. To test this hypothesis, we injected diploid- and tetraploid-sorted TBP ARPE cells with or without stable ADAR1 knockdown under the skin of immune-compromised NMRI mice, which lack T-cells, and monitored tumor outgrowth over time. Diploid TBP ARPE reached the ethically tolerated tumor size of 1.5 cm^3^ relatively fast, on average after 21 weeks (Figure 7A, upper panel left). In agreement with their *in vitro* behavior in non-permissive culturing conditions, tetraploid-sorted cells showed delayed tumor outgrowth, reaching the maximally tolerated size after on average 28 weeks (Figure 7A, lower panel left) and thus extending survival (Figure 7B). ADAR1 knockdown in both tetraploid-sorted and diploid-sorted TBP ARPE cells delayed tumor growth, taking on average 35 weeks and 33 weeks, respectively, to reach the maximum size (Figure 7A, lower panel right, upper panel right, Figure 7B). The latter seems in contrast to our serum starvation experiments *in vitro* where ADAR1 depletion did not affect mitogen-independent proliferation of diploid cells (Figure 4C). However, the *in vitro* experiments shown in Figure 4C were short term (2 weeks), while upon long-term culturing of diploid ADAR1 knockdown TBP cells, tetraploid cells started to accumulate only after approximately 6 weeks (Figure 5A, B). We therefore envisage that also *in vivo* ADAR1 depletion caused tetraploidization and that selective pressure against genome-duplicated cells retarded tumor growth. To test this possibility, we cultured all tumor material *ex vivo* for 2–4 days in the presence of mitogens and, taking advantage of the presence of FUCCI constructs in the injected cells, measured the DNA content in G1 cells by flow cytometry as explained in Figure 2. As expected, ADAR1-proficient diploid cells remained diploid (Figure 7C, light grey bars). ADAR1-depleted diploid tumors showed variable levels of tetraploid karyotypes, in one case almost reaching 50% (Figure 7C, left panel). The fraction of tetraploid cells in tumors originating from ADAR1-proficient tetraploid-sorted cultures showed a variable but clear reduction, while tetraploidy was almost lost when ADAR1 was depleted (Figure 7C, right panel). These results show that tetraploidization not only conferred a proliferative impediment to TBP cells cultured in serum-deprived conditions *in vitro* but also when grown as tumors *in vivo*. The absence of ADAR1 may aggravate this proliferative defect likely because of on-going polyploidization leading to chromosome abundance incompatible with proliferation.

## Discussion

In this study we identified an unexpected consequence of ADAR1 depletion: whole genome duplication. We demonstrated that both the catalytic- and Zα domain of ADARp150 are essential to prevent tetraploidization. Absence of functional ADARp150 induced expression of Cyclin B3 which was causative for an increased frequency of mitotic catastrophe and the accumulation of tetraploid cells. We identified this mitotic requirement for ADARp150 in G1/S-checkpoint defective, apoptosis-resistant, p53 defective cells (TBP cells), cultured in the absence of mitogens and suffering from DNA replication stress. However, also in growth-promoting conditions ADAR1-depleted TBP cells accumulated to near-complete tetraploidy, without showing a discernable proliferative defect. In contrast, in growth-restricting conditions, tetraploidy arising from ADARp150 depletion severely hindered proliferation. But why is division of tetraploid cells hindered in mitogen-deprived conditions, i.e. under conditions of perturbed DNA replication? We envisage that mitogen-deprived cells with higher DNA content may suffer more from replication-induced DNA damage, *e.g*. because of a higher chance of mitotic catastrophe. This may explain the reduced colony formation of mitogen-deprived, ADAR1-proficient TBP ARPE cells shown in Figure 4C. This effect may be exacerbated by high Cyclin B3 activity in the absence of ADAR1 that could prematurely drive cells with damaged DNA into catastrophic M-phase.

In several isogenic cell lines, ADAR1 deficiency only yielded tetraploid karyotypes upon concomitant p53 inactivation. Presumably, p53 activation by WGD itself or certain types of DNA damage that may promote WGD, results in cell cycle arrest or apoptosis, thereby preventing the accumulation of tetraploid cells (37,38). The requirement for p53 deficiency could explain why a mitotic role of ADAR1 has been underappreciated, albeit not fully ignored.

Earlier studies have found a link between ADAR1 and mitotic progression (7,39,40). Shiromoto *et al.* observed an increase in mitotic abnormalities such as the formation of micro- and multi-nuclei and anaphase bridges upon ADAR1 depletion in HeLa cells, which was associated with an accumulation of R-loops at telomeres due to shortage of ADARp110 activity (7). In contrast, the mitotic defects in ADAR1-depleted cells we describe here appeared to result from an aberrant induction of Cyclin B3, which was abolished upon re-introduction of ADARp150. None of the other A-, B-, D- or E-type cyclins was induced. B-type cyclins are critical regulators of mitotic entry and are degraded in a timely manner during mitosis (41). Cyclin B1 is mainly localized in the cytoplasm during interphase and translocates to the nucleus during prophase to promote nuclear envelope breakdown and mitotic entry (42,43). Inactivation of the spindle assembly checkpoint leads to activation of APC/C^CDC20^ which targets Cyclin B1 for proteolytic destruction at anaphase onset (44). Cyclin B3 is a late degrading B-type cyclin and is degraded during anaphase (35). Studies have proposed that Cyclin B3 promotes APC/C^CDC20^ activity and anaphase initiation (33,34). Aberrant induction of Cyclin B3 could disturb the metaphase to anaphase transition and mitotic exit thus causing the mitotic failures that we observed upon ADARp150 depletion. Indeed, expression of a non-degradable form of Cyclin B3 has been shown to induce mitotic arrest late in anaphase (36). How ADARp150 affects Cyclin B3 expression and whether this occurs in a particular cell cycle phase remains an open question.

A role of ADARp150 in suppressing IFN signaling in response to endogenous transcripts has been well established (45–49). Somewhat unexpectedly, we did not observe increased IFN signaling upon ADARp150 depletion. In fact, total RNA sequencing indicated that IFN signaling was *reduced* upon ADAR1 knockdown. Possibly, TBP ARPE cells have an impairment in IFN signaling, which is supported by the marginal induction of IFNAR1 expression upon treatment with the immunostimulant poly(IC) (Supplementary Figure S2B). Alternatively, ADAR1 knockdown may force a new, attenuated level of IFN signaling. Anyway, the lack of IFN signaling upon ADAR1 depletion in the TBP cells we used may have allowed us to find the mitotic defect that underlies ADAR1 dependency of mitogen-starved TBP cells.

ADARp150 depletion and the resulting tetraploidy also significantly delayed tumor outgrowth *in vivo*. The inability of tetraploid cells to proliferate *in vivo* as well as in non-permissive culturing conditions *in vitro* is suggestive for a non-permissive microenvironment that impacts tumor outgrowth. While such environment may be transient and differ spatially within tumors depending on the vascularization, several studies have provided evidence for a growth-inhibiting tumor microenvironment (50,51).

ADAR1 has been proposed as a therapeutic target to enhance the efficacy of immunotherapy (52–55). Collectively, these studies showed that ablation of ADAR1 results in activation of pattern recognition receptors, thereby activating IFN signaling. The whole genome duplications that we observed upon ADAR1 depletion represent another layer of ADAR1 biology. The relevance of our results for AGS biology and ADAR1 as a target for immunotherapy remains to be determined. ADAR1 has been found overexpressed in several tumor types (29,56,57). This has been mostly attributed to the canonical function of ADAR1 as a negative regulator of IFN signaling. Our studies provide another perspective, preventing whole genome duplication.

## Supplementary Material

gkae700_Supplemental_Files

## Data Availability

Raw and normalized read-counts of the RNA-sequencing experiment have been deposited in GEO (https://www.ncbi.nlm.nih.gov/geo/) under accession number GSE262277.
