# Supplementary material for: ADARp150 counteracts whole genome duplication
Source: Nucleic Acids Res. 2024 Aug 27;52(17):10370–84. doi: 10.1093/nar/gkae700 (PMC11417406; doi:10.1093/nar/gkae700)

Supplementary legends

Supplementary Figure 1 (A) Protein levels of ADARp110 and ADARp150 of TBP MEFs. γ-tubulin was used as a loading control. **(B-D)** Protein levels of p130, pRB and p107 (B), Bcl2 (C) and p53 with or without 20 Gray irradiation (D) in WT and TBP ARPE cell lines. γ-tubulin was used as a loading control. **(E)** Growth curves of TBP ARPE cells cultured with or without 10% FCS in the presence or absence of 10 nM CHK1 inhibitor UCN01. Growth curves were obtained by using the IncuCyte. **(F)** Protein levels of ADARp110 and ADARp150 in TBP ARPE cells transduced with a non-targeting (NT) or *ADAR1* shRNA. γ-tubulin was used as a loading control. **(G)** Replication fork speed of TBP ARPE cells transduced with the indicated shRNA, cultured in the presence (+) or absence (-) of 10% FCS. Ongoing replication forks were used to calculate fork speed divided over three independent experiments. Dots represent the speed of individual replication forks. Sample sizes: NT shRNA +FCS n=191, -FCS n=494, *ADAR1* shRNA +FCS n=491, -FCS n=486. Black horizontal lines indicate the mean replication fork speed. Asterisks represent adjusted p-value of an ordinary one-way ANOVA test (Tukey's multiple comparison test) (****p-value < 0.0001). **(H)** Quantification of the level of origin firing of TBP ARPE cells transduced with a non-targeting (NT) or *ADAR1* shRNA, cultured with (+) or without (-) 10% FCS. The percentage of origins fired during the 1st or 2nd labeling is shown relative to all labelled tracks. Dots represent the average level of origin firing in 3 independent experiments. Error bars indicate the standard deviation. Statistical significance was assessed using an ordinary one-way ANOVA test (Šidák multiple comparison test). ns = non-significant. Comparisons to NT shRNA +FCS: NT shRNA -FCS p=0.00754, ADAR1 shRNA +FCS p=0.6196, ADAR1 shRNA -FCS p=0.0800. **(I)** Tail moments of TBP ARPE cells transduced with a NT or *ADAR1* shRNA, cultured with (+) or without (-) 10% FCS. Dots represents the tail moment of individual cells. Black horizontal lines indicate the mean tail moment. Representative image of three independent experiments. Sample sizes: NT shRNA +FCS n=50, -FCS n=50, *ADAR1* shRNA +FCS n=50, -FCS n=54. Asterisks represent adjusted p-value of an ordinary one-way ANOVA test (Tukey's multiple comparison test). ns = non-significant.

Supplementary Figure 2. (A) Population doublings (h) of non-targeting (NT) and *ADAR1* shRNA transduced TBP ARPE cells with indicated concomitant gene knockout clones grown in unperturbed culturing conditions (+10% FCS) and passed before reaching confluency. Dots

represent 2-9 independent measurements. Error bars indicate standard deviation. **(B)** IFNAR1 protein levels in indicated TBP ARPE cells from (A) treated with or without 1.5 µg/mL polyIC. γ-tubulin was used as a loading control. **(C)** MDA-5 (*IFIH1*) protein levels in indicated TBP ARPE cells from (A) treated with or without 100 ng/mL IFNα 2b for 3 days. γ-tubulin was used as a loading control. **(D)** LGP2 (*DHX58*) protein levels of indicated TBP ARPE cells from (A) treated with or without 1.5 µg/mL polyIC. γ-tubulin was used as a loading control. **(E)** RIG-1 (*RIG-1*) protein levels of indicated TBP ARPE cells from (A) treated with or without 1.5 µg/mL polyIC. γ-tubulin was used as a loading control. **(F)** PKR (*EIF2AK2*) protein levels of indicated TBP ARPE cells from (A). γ-tubulin was used as a loading control. **(G)** RNAseL (*RNASEL)* protein levels of indicated TBP ARPE cells from (A). γ-tubulin was used as a loading control. **(H)** ZBP1 (*ZBP1*) protein levels of indicated TBP ARPE cells from (A). γ-tubulin was used as a loading control. **(I)** Representative images of serum-starved TBP ARPE cells transduced with a non-targeting (NT) or *ADAR1* shRNA with or without concomitant gene knockout of *IFIH1*, *IFNAR1*, *RIG-1*, *RNASEL*, *EIF2AK2* or *ZBP1* or treated with 0.5 or 1.0 µM of integrated stress response inhibitor (ISRIB). 25.000 cells were grown in the absence of serum for 14 days in 6-well plates. **(J)** Ingenuity Pathway Analysis of RNA sequencing data of pathways related to the canonical function of ADARp150. Rows indicate relative comparisons of ADAR1 knockdown TBP ARPE cells reconstituted with vector only (pmGFP) or ADARp150 (pmGFP-ADARp150) compared to TBP ARPE cells transduced with a non-targeting (NT) shRNA.

Supplementary Figure 3. **(A)** Protein levels of ADARp110 and ADARp150 in ARPE cells transduced with NT or *ADAR1* shRNA. γ-tubulin was used as a loading control. **(B)** Percentage of indicated ARPE cells in G1 phase with a DNA content larger than 2n, as identified by the method described in Figure 2C. Dots indicate independent measurements. Asterisks represent adjusted p-value of an ordinary one-way ANOVA test (Tukey's multiple comparison test) (****p-value < 0.0001) **(C)** Protein levels of ADARp110 and ADARp150 in HCT116 cells transduced with NT or *ADAR1* shRNA. γ-tubulin was used as a loading control. **(D)** Number of chromosomes in individual chromosome spreads of wild-type or p53$^{-/-}$ HCT116 cells with (blue) or without (grey) ADAR1 knockdown. Dots represent chromosome numbers in individual chromosome spreads. Dashed horizontal line represents a cut-off of n=50 to quantify the fraction of diploid cells. **(E)** Protein levels of ADARp110 and ADARp150 in MCF7 cells transduced with NT or *ADAR1* shRNA. γ-tubulin was used as a loading control. **(F)**

Number of chromosomes in individual chromosome spreads of wild-type or p53$^{-/-}$ MCF7 cells with (blue) or without (grey) ADAR1 knockdown. Dots represent chromosome numbers in individual chromosome spreads. **(G)** Number of chromosomes in individual chromosome spreads of ADAR1 knockdown TBP ARPE cells with control (NT sgRNA) or concomitant *IFNAR1* gene knockout. Dashed horizontal line represents a cut-off of n=50 to quantify the fraction of diploid cells.

Supplementary Figure 4. (A) Ingenuity Pathway Analysis of RNA sequencing data of selected pathways related to cell cycle regulation and mitosis. Rows indicate relative comparisons of ADAR1 knockdown TBP ARPE cells reconstituted with vector only (pmGFP) or ADARp150 (pmGFP-ADARp150) compared to TBP ARPE cells transduced with a non-targeting (NT) shRNA. Colors indicate the log2-fold changes. **(B)** Relative expression levels of the genes included in the ingenuity pathway “mitotic roles of polo-like kinase”. Colors indicate the log2-fold change.

Supplementary Figure 1

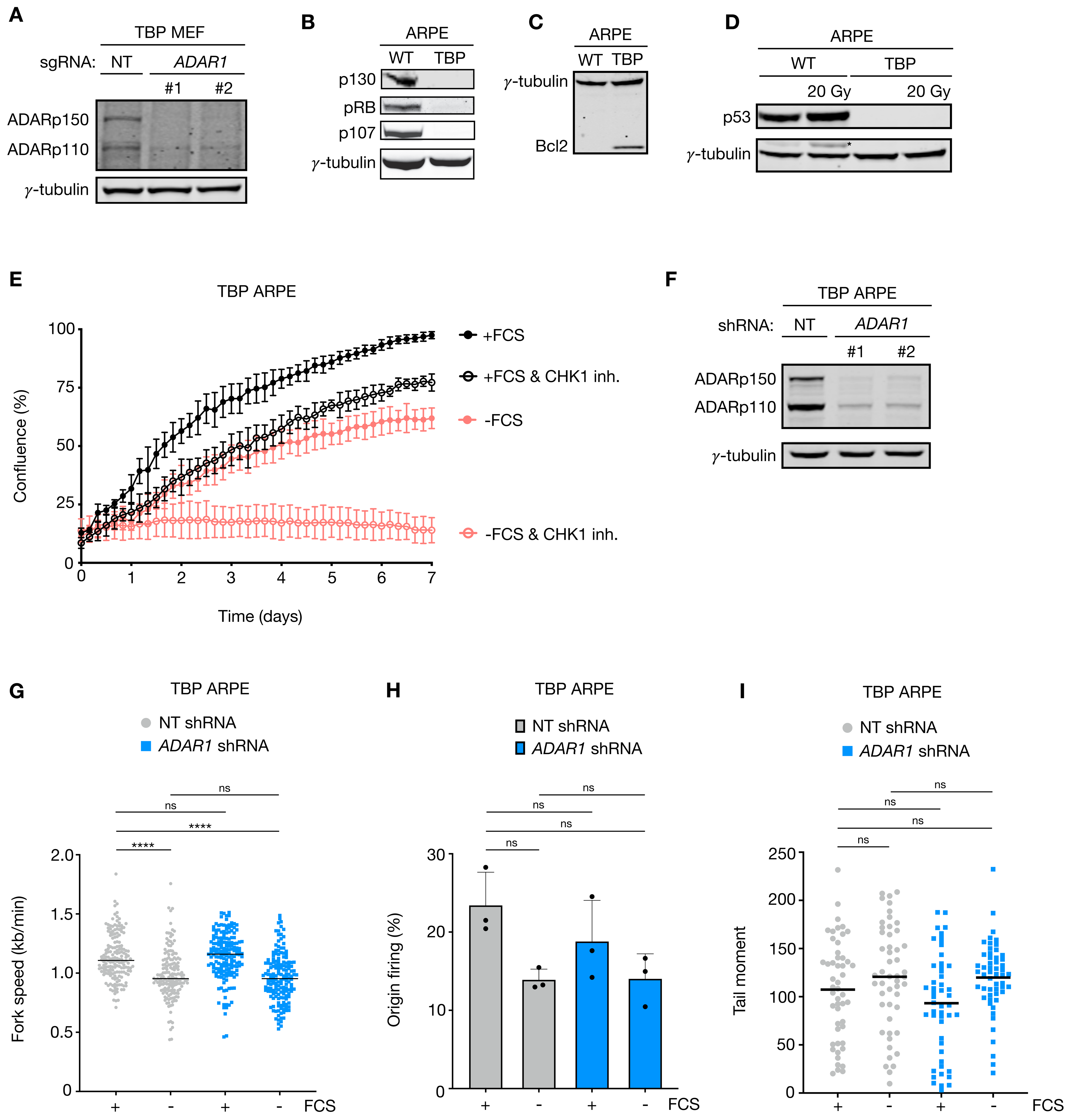

Supplementary Figure 2

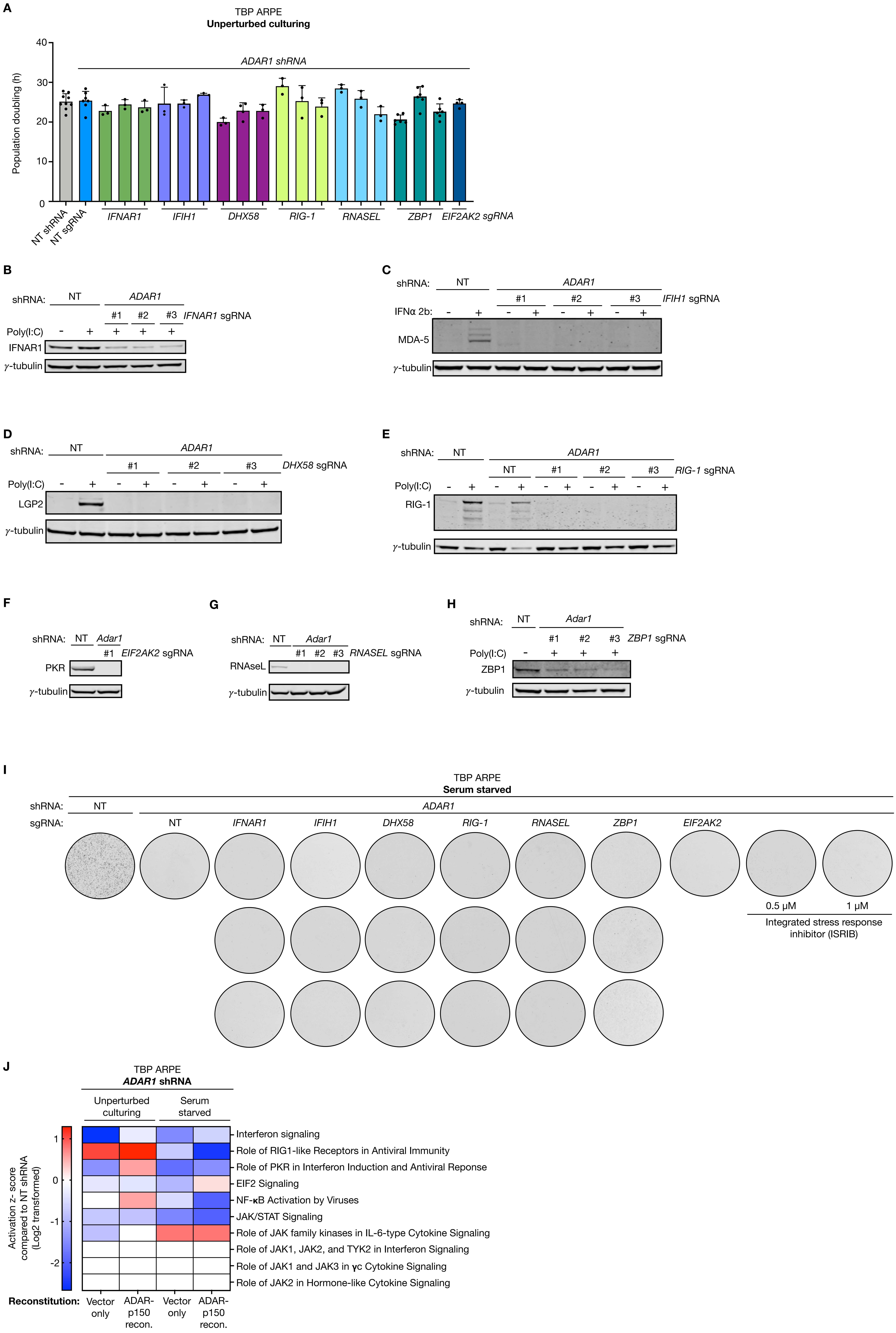

Supplementary Figure 3

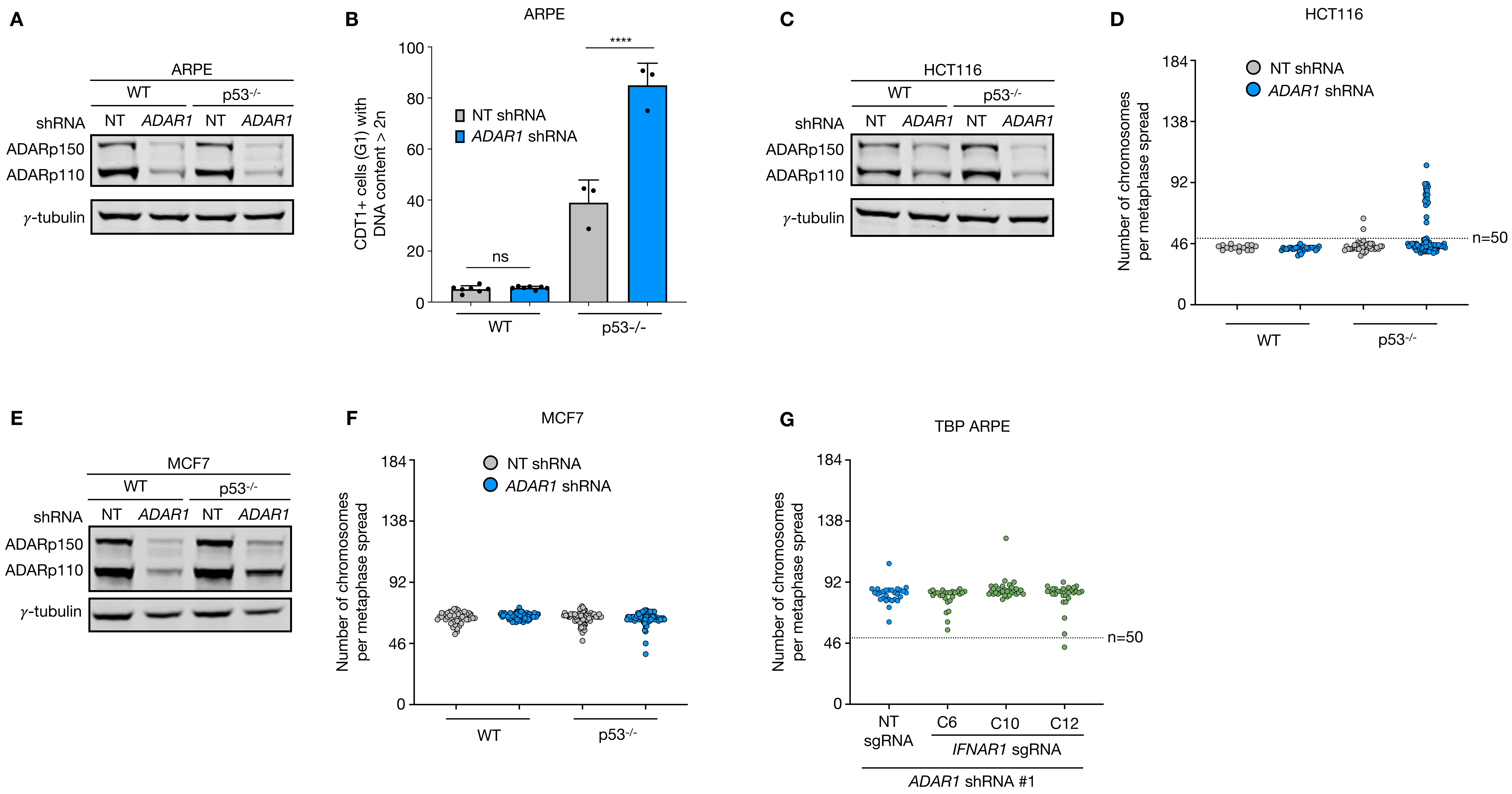

Supplementary Figure 4

A

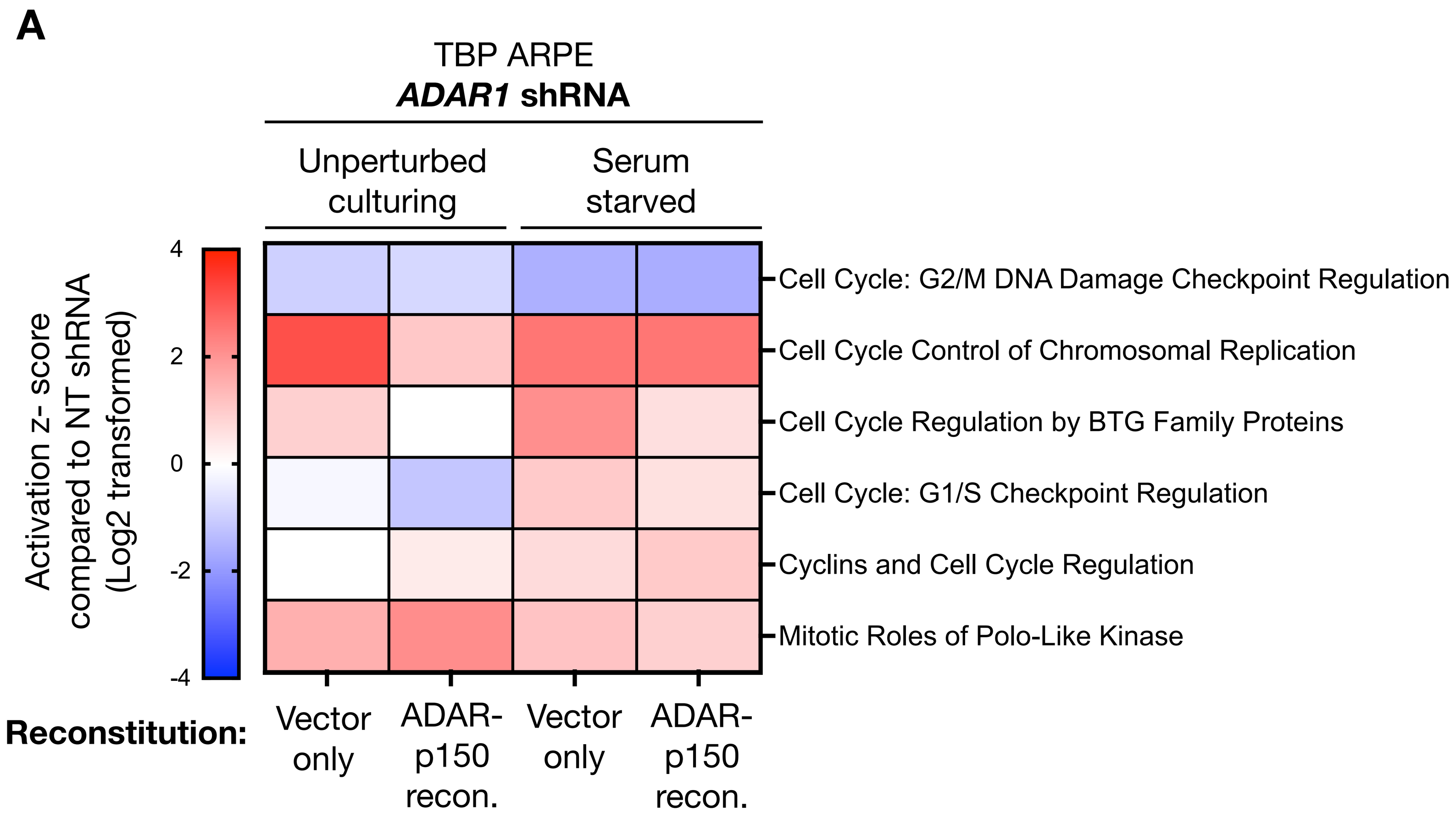

B

Mitotic Roles of Polo-Like Kinase

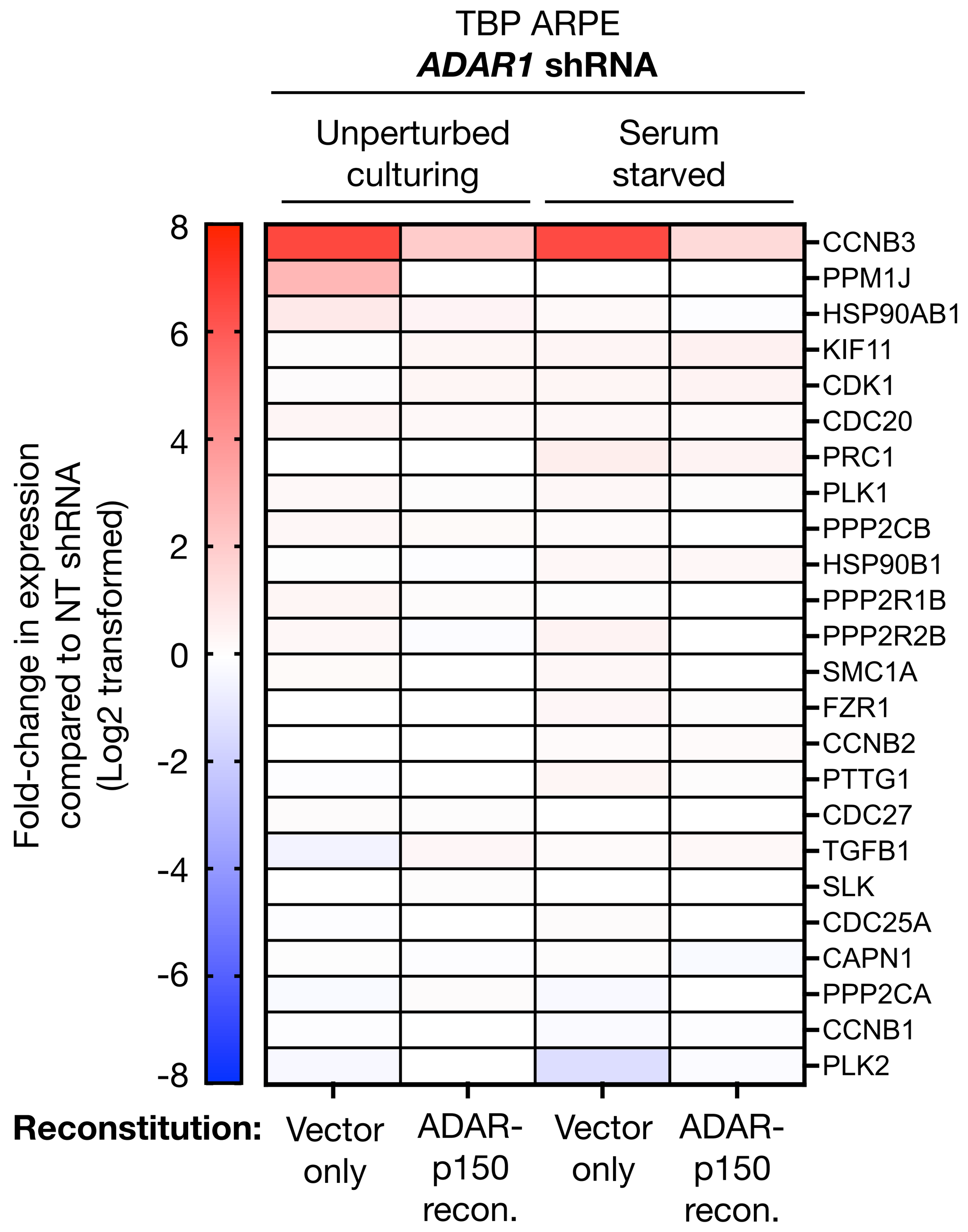

Supplement: gkae700_Supplemental_Files [file gkae700_supplemental_files.zip › gkae700 ADARp150 counteracts whole genome duplication - Supplementary Figures.pdf]
